# An Overview of Chitosan Nanoparticles and Its Application in Non-Parenteral Drug Delivery

**DOI:** 10.3390/pharmaceutics9040053

**Published:** 2017-11-20

**Authors:** Munawar A. Mohammed, Jaweria T. M. Syeda, Kishor M. Wasan, Ellen K. Wasan

**Affiliations:** College of Pharmacy and Nutrition, University of Saskatchewan, Saskatoon, SK S7N 2Z4, Canada; mum495@mail.usask.ca (M.A.M.); jaweria.syeda@usask.ca (J.T.M.S.); kishor.wasan@usask.ca (K.M.W.)

**Keywords:** chitosan, mucoadhesive, polymeric nanoparticles, sustained release, oral drug delivery

## Abstract

The focus of this review is to provide an overview of the chitosan based nanoparticles for various non-parenteral applications and also to put a spotlight on current research including sustained release and mucoadhesive chitosan dosage forms. Chitosan is a biodegradable, biocompatible polymer regarded as safe for human dietary use and approved for wound dressing applications. Chitosan has been used as a carrier in polymeric nanoparticles for drug delivery through various routes of administration. Chitosan has chemical functional groups that can be modified to achieve specific goals, making it a polymer with a tremendous range of potential applications. Nanoparticles (NP) prepared with chitosan and chitosan derivatives typically possess a positive surface charge and mucoadhesive properties such that can adhere to mucus membranes and release the drug payload in a sustained release manner. Chitosan-based NP have various applications in non-parenteral drug delivery for the treatment of cancer, gastrointestinal diseases, pulmonary diseases, drug delivery to the brain and ocular infections which will be exemplified in this review. Chitosan shows low toxicity both in vitro and some in vivo models. This review explores recent research on chitosan based NP for non-parenteral drug delivery, chitosan properties, modification, toxicity, pharmacokinetics and preclinical studies.

## 1. Introduction

The mucosal route is gaining attention for noninvasive drug delivery via the oral, nasal, pulmonary or vaginal routes [1]. At the same time, nanoparticle technology has also come to the forefront as a viable drug delivery strategy, presenting opportunities for controlled release, protection of active components from enzymatic or environmental degradation and localized retention. Nanoparticle fabrication methods are readily scalable and applicable to a broad range of drugs. Of all the nanoparticle drug delivery approaches, polymeric nanoparticles have gained significant importance as they are biodegradable, biocompatible and because formulation methods are more widely available; the range of applications has been expanding to include a variety of chemical drug classes and dosage forms [2]. Chitosan-based NP are particularly appropriate for the mucosal route, with their low toxicity, mucoadhesion and tunable physical properties. Examples will be given of chitosan-based nanoparticles used for the treatment of cancer, gastrointestinal diseases, pulmonary diseases, drug delivery to the brain and ocular infections. Recent research on chitosan-based NP for nonparenteral drug delivery is based on the field’s expanding understanding of chitosan properties and methods of chemical or physical modification, which are applied to the optimization of nanoparticle drug loading and release features. We will also discuss the current understanding of in vitro and in vivo toxicity and the effect of chitosan nanoparticle formulation on drug pharmacokinetics in preclinical studies.

### Chitosan

**Figure 1:**
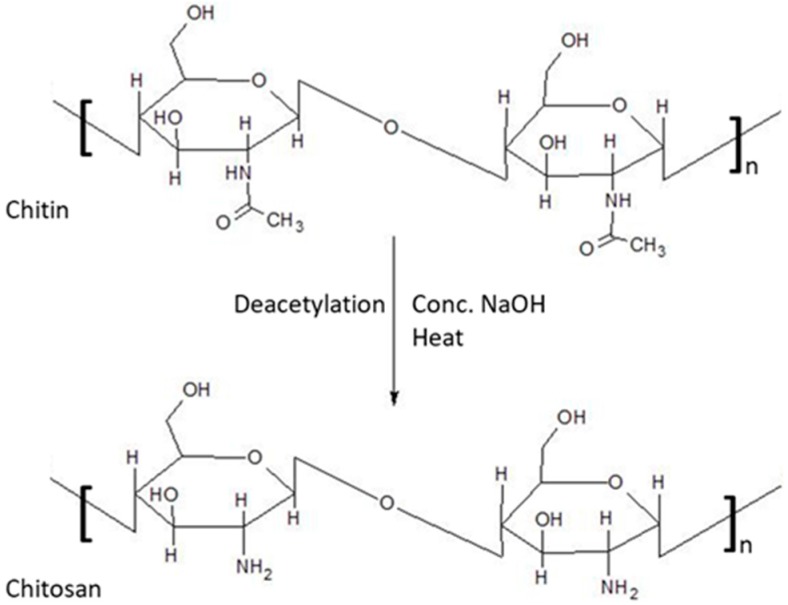
Deacetylation of chitin to chitosan.

Chitosan is the most important derivative of chitin, produced by removing the acetate moiety from chitin as shown in Figure 1.

It is derived from crustacean shells such as those from prawns or crabs, as well as from the cell walls of fungi. It is a naturally occurring polysaccharide, cationic, highly basic, mucoadhesive biocompatible polymer and approved by the U.S. FDA for tissue engineering and drug delivery. Chitin from natural sources is found bound to proteins and minerals, which must be removed prior to preparation of chitosan, though processes of acidification and alkalization. Purified chitin is then *N*-deacetylated to chitosan. This process can be modified to control the end product properties [including molecular weight and pKa (6–7.5)] [3,4] by controlling the degree of deacetylation with factors such as reaction conditions (concentration, ratios of chitin to alkali, temperature), chitin source and extent of the reaction, for example. While these chemical processes are well in hand for industrial processors, research is ongoing to more fully develop scalable biological, enzymatic or hybrid methods such as those using microorganisms [5]. It will be interesting to see how these approaches will be used to manufacture specific types of deacetylated chitosan and whether these bioprocesses will have any environmental advantage in the future.

**Figure 2:**
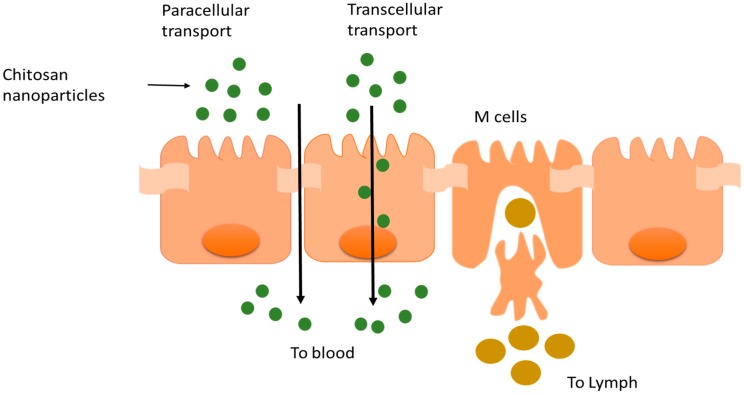
Schematic illustration of the presumed mechanism of transcellular and paracellular transport of chitosan NP across the epithelium.

Chitosan acts a penetration enhancer by opening the tight junctions of the epithelium. Chitosan facilitates both paracellular and transcellular transport of drugs as indicated in Figure 2. Chitosan interacts with mucus (negatively charged) to form a complex by ionic or hydrogen bonding as well as though hydrophobic interactions. The pKa of the primary amine of chitosan is ~6.5, depending on the degree of *N*-deacetylation. This group also contributes to the solubility of chitosan in acidic pH environments and the partial neutralization of this primary amine may also explain why chitosan has been reported to aggregate at neutral to high pH [6]. However, it should be noted that while this may be the tendency for chitosan with fraction of acetylated units <0.4 and medium/high molecular weight, chitosan with acetylation degrees in the range 40–60% are well known as being soluble even at physiological pH [4]. Thus, the nanoparticle formulator must carefully match the desired chemical and physical properties of the chitosan, as well as the anticipated biological environment, with the chitosan processing method.

## 2. Modification of Chitosan

The chitosan backbone can be modified to alter properties such as solubility, mucoadhesion and stability as discussed throughout this paper for specific applications. Both the -NH_2_ and -OH groups of chitosan are the active sites for modification. Some of the commonly used techniques described below for preparing chitosan polymers are: blending, graft co-polymerization and curing [7]. Blending involves the simple mixing of two or more polymers. Graft co-polymerization involves the covalent bonding of polymers, while curing converts the polymers into a solidified mass by formation of three-dimensional bonds within the polymer mass by means of thermal, electrochemical or ultraviolet radiation processing methods.

### 2.1. Physical Modification

Physical modification is achieved by blending, which involves the physical mixing of two or more polymers. It is one of the oldest and easiest ways of modifying polymers. The quality and performance of the blend can be modified depending on the ratios of the polymers which are being mixed. Blending is the most economical technique by which the polymer properties can be tailored for specific applications [8].

Some of the common hydrophilic polymers that can be blended with chitosan to achieve oral drug delivery are poly (vinyl alcohol) (PVA), poly (vinyl pyrrolidone) (PVP) and poly (ethyl oxide) (PEO). Blending of chitosan and PVA improves the mechanical (tensile strength) and barrier properties (water vapor permeability) of chitosan films [9]. The intermolecular interactions between chitosan and PVA result in blends of PVA-chitosan that show improved mechanical properties (tensile strength) of chitosan films for controlled drug delivery [10]. An example of physical modification in controlled drug delivery is represented by amoxicillin formulated with a crosslinked chitosan/PVP blend with glutaraldehyde to form a semi-interpenetrating polymer (semi-IPN) [11]. The semi-IPN is formed because of the protonation of the amino group of chitosan, as confirmed by Fourier transform infrared spectroscopy (FTIR). Additional methods used for characterization of chitosan blends other than FTIR are: differential scanning calorimetry (DSC) [12], X-ray diffraction (XRD) [13], FTIR spectroscopy and rheology [14], enabling an understanding of the effects of processing on bonding and flow properties.

### 2.2. Chemical Modification

Chemical modification is achieved by altering the functional groups in a compound. Chemical modification can be done by several ways which include: chemical, radiation, photochemical, plasma-induced and enzymatic grafting methods [7]. Chemical modification of chitosan results in the formation of several derivatives such as quaternized chitosan, thiolated chitosan, carboxylated chitosan, amphiphilic chitosan, chitosan with chelating agents, PEGylated chitosan and lactose-modified chitosan. The primary amine (-NH_2_) groups of chitosan provide a reaction site for chemical modification to achieve various pharmaceutical applications [7], reacting with sulphates, citrates and phosphates [15], which can enhance the stability and drug encapsulation efficiency [16]. For example, to improve the solubility of chitosan in intestinal media, *N*-trimethyl chitosan chloride (TMC), a quaternized chitosan, has been produced [17]. The two forms TMC, TMC 40 and TMC 60, enhance the intestinal permeation of hydrophilic macromolecular drugs. The mucoadhesiveness of chitosan has been further enhanced by formulating NP with thiolated chitosan [18]. Quaternization of chitosan forms several derivatives such as trimethyl (TMC), dimethylethyl (DMEC), diethylmethyl (DEMC) and triethyl chitosan (TEC). Quaternization of chitosan aids in the opening of tight junctions and improving the permeability of insulin across Caco-2 cells [19]. Chitosan-thioglycolic acid, chitosan-cysteine, chitosan-glutathione, chitosan-thioethylamidine are some of the thiolated chitosan derivatives presently in use. TMC-cysteine based NPs have shown significantly higher mucoadhesion and permeation compared to TMC NPs [20]. Grafting carboxylated chitosan with poly (methyl methacrylate) helps achieve pH-sensitive properties. The NPs made with the grafted polymer and insulin have shown very minimal drug release in simulated gastric fluid and an instant release in simulated intestinal fluid [21]. A pH sensitive polymer gel can be prepared by chemically linking d,l-lactic acid with -NH_2_ groups of the chitosan for an application in the drug delivery to the different regions of the gastrointestinal tract (GIT) [22]. Lactose modification of the chitosan backbone (1-deoxylactit-1-yl chitosan) has been used in combination with the polyvalent ion tripolyphosphate (TPP) to form colloidal coacervates though polyionic interactions, forming highly uniform and small (200 nm diameter) nanoparticles [23,24].

## 3. Drug Release from Chitosan Nanoparticles

**Figure 3:**
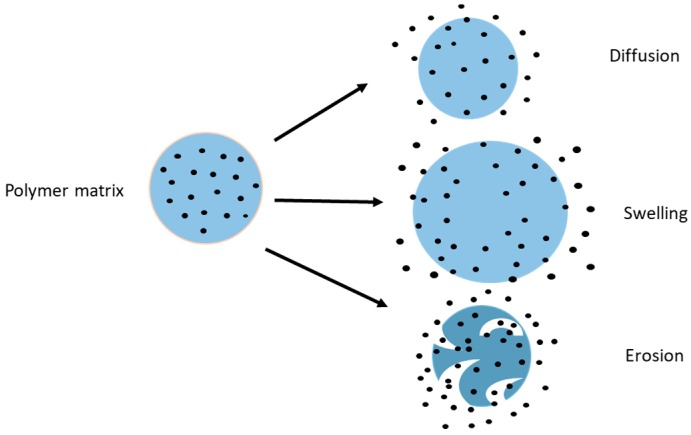
Diagram representing the possible mechanisms of drug release by diffusion, swelling and erosion of polymer (chitosan) matrix.

There are several mechanisms which govern drug release from chitosan nanoparticles such as: swelling of the polymer [25], diffusion of the adsorbed drug, drug diffusion through the polymeric matrix, polymer erosion or degradation and a combination of both erosion and degradation [26] as represented in Figure 3. The initial burst release from the chitosan nanoparticles is either because of swelling of the polymer, creating pores, or diffusion of the drug from the surface of the polymer [27]. Chitosan nanoparticles also exhibit a pH-dependent drug release because of the solubility of chitosan [28]. Chitosan derivatives alter the release of drug from the NP, affording tunable drug release [29] and impacting the pharmacokinetic profile of the loaded drug.

In diffusion-controlled release, the drug permeates through the interior of the polymeric matrix to the surrounding medium. Polymer chains form the diffusion barrier making it difficult for the drug to pass through and this barrier serves as the rate-limiting membrane for drug release. Diffusion may also be associated with polymer swelling or erosion. The mathematical representation of diffusion is given by Fick’s law of diffusion.

(1) $$F=-D\frac{\partial c}{\partial x}$$

where, *F* is the rate of transfer per unit area of section (flux), *c* is the concentration of the drug and *D* is the diffusion coefficient (diffusivity). To derive the parameters of Fick’s law, there are few assumptions to be made such as: pseudo-steady state is maintained during drug release, the diameter of the drug particles is less than the average distance of drug diffusion through the polymeric matrix and sink conditions are always provided by the medium surrounding the nanoparticles [30].

The swelling of the polymer is characterized by the imbibition of water into the polymer until the polymer dissolves. This drug release mechanism is characterized by the solubility of the polymer in water, or the surrounding biological medium. When the polymer encounters the surrounding medium and swelling commences, the polymer chains detangle. This is followed by drug release from that region of the polymer matrix. Generally, the hydrophilicity of the polymer, the polymer swelling rate and the density of the polymer chains play a key role in the drug release profile [31]. Subsequently, this will affect the rate of drug absorption from the site of delivery in vivo, as it will affect the rate at which drug is available for membrane transport or cellular uptake.

Erosion and degradation of polymers are interrelated features. Sometimes, degradation of the polymer may cause subsequent physical erosion as bonds break. Erosion of polymers is a complex phenomenon as it involves swelling, diffusion and dissolution. Erosion occurs in two ways: homogenous and heterogeneous. Homogenous erosion is erosion of the polymer at the same rate throughout the matrix whereas heterogeneous erosion is erosion of the polymer from the surface towards the inner core. Polymer degradation may be due to the surrounding media or the presence of enzymes. The degradation of the polymer also depends on the pH of the surrounding media, the copolymer composition and water uptake by the polymer. Drug release depends on the type of polymer and internal bonding, any additives (chitosan derivatives), as well as the shape and size of the nanoparticles as this reflects surface area and free energy [32].

Generally, drug release from the chitosan nanoparticles is similar to that of PLGA (Poly(d,l -lactide-*co*-glycolide)) nanoparticles [33] but the drug release from chitosan nanoparticles is more pH-dependent [34]. As an example, exendin-4 loaded PLGA and chitosan-PLGA nanoparticles have been studied. Exendin-4 is generally used for the treatment of type-2 diabetes. The NPs were evaluated for transmembrane permeability studies in MDCK (Madin-Darby Canine Kidney) cells and in an in vivo study in male Wistar rats. The in vitro transmembrane permeability studies revealed that exendin-4 was transported across the cell layer by active transport when formulated as PLGA or chitosan-PLGA NP compared to the free drug solution. The permeability coefficient (P_app_) with PLGA and chitosan-PLGA NP was 1.52 × 10^−6^ and 2.5–3.0 × 10^−6^, respectively, significantly greater than exendin-4 solution alone. Some of the PLGA NPs that enter the cell are trafficked into endosomes and suffer degradation while some reach the basolateral membrane via the trans-Golgi pathway. Similar mechanisms occur with chitosan-PLGA NPs, however, due to their positive charge electrostatic interactions occur with the negatively charged cell membrane resulting in a higher P_app_. The in vivo study revealed higher plasma drug levels and longer retention times of exendin-4 when administered as chitosan-PLGA NP compared to PLGA NP [35].

## 4. Chitosan in Oral Drug Delivery

Oral drug delivery certainly is the most convenient route of drug administration because of the ease of administration. But there are several challenges in achieving oral delivery such as varying pH (highly acidic stomach), the presence of enzymes, first-pass effect in the liver and the intestinal barrier to drug absorption. The above challenges limit the drug from entering the systemic circulation thereby reducing oral bioavailability [36]. Nanoparticle technology is an increasingly exploited formulation technique to overcome the limitations of oral drug delivery [7,37]. NPs have several advantages such as small particle size, large surface area and potentially a modifiable surface. Small particle size is well known to increase the dissolution rate of drugs. Besides these advantages, NPs can increase the gastrointestinal tract stability of acid-labile drugs compared to other drug delivery systems such as liposomes and lipid based systems [38]. Chitosan can be formulated as polymeric nanoparticles for various applications in oral drug delivery as explained below with several examples.

Catechin and epigallocatechin are flavonoids present in green tea and are strong antioxidants. These undergo degradation in intestinal fluid and are poorly absorbed across intestinal membranes. The intestinal absorption of catechin and epigallocatechin gallate can be improved by encapsulating them in chitosan nanoparticles [39]. Tamoxifen, an anti-cancer drug, is slightly water soluble and a good candidate for oral cancer drug delivery. Permeation of tamoxifen across the intestinal epithelium was increased by formulating tamoxifen into lecithin-chitosan nanoparticles [40]. The NPs are mucoadhesive and increase the permeation of tamoxifen by the paracellular pathway. Feng et al. have also reported a potential oral delivery strategy for anti-cancer drugs. They have prepared nanoparticles of doxorubicin hydrochloride (DOX) with chitosan and carboxymethyl chitosan. These nanostructures were found to enhance the intestinal absorption of DOX throughout the small intestine [41]. Alendronate sodium is used in the treatment of osteoporosis and suffers from low oral bioavailability and gastrointestinal side effects. High NP encapsulation efficiency of alendronate sodium was achieved by formulating chitosan nanoparticles via an ion gelation technique. Drug release was clearly pH-dependent; in 0.1N HCl, almost 80% of the drug was released within 60 min while in PBS (pH 6.8) a maximum of 40% of the drug was released over 4 h, suggesting that factors other than chitosan’s pH solubility profile influenced drug release in this case and that optimization is multifactorial [28]. This highlights the importance of examining the degree of surface coverage of the nanoparticles with chitosan and of performing ongoing dissolution studies in biorelevant media during formulation development. For effective sustained delivery of sunitinib, a tyrosine kinase inhibitor, chitosan NPs were prepared by an ion crosslinking method. The encapsulation efficiency of the NPs was 98% and sustained drug release was achieved up to 72 h [42]. The harsh conditions of the GIT denature proteins such as insulin when administered orally, yet oral insulin is a highly desired goal. In one example of insulin-loaded chitosan NPs the chitosan was crosslinked with tripolyphosphate. The particle size was reduced by this crosslinking process and the stability of the NPs was increased by freeze drying. NP uptake was significant in the intestine epithelium; unfortunately, however, the NPs were unstable in gastric pH [43] and further efforts to fabricate stable oral insulin NP are still needed. Bay 41-4109, an active inhibitor of hepatitis B virus was formulated as chitosan NPs to improve drug solubility and oral bioavailability. The cytotoxicity of Bay 41-4109-chitosan NP was found to be negligible and drug uptake was higher from the NPs, which was attributed to the positive charge from the chitosan [44]. Table 1 provides detailed insight into the extensive range of applications of chitosan NP for oral drug delivery.

Oral delivery of vaccines is critical as the antigens degrade in GIT hindering their reaching Peyer’s patches, the gastrointestinal lymphoid tissue. Moreover, vaccines containing chitosan NP can only be prepared by methods that avoid organic solvents as the organic solvents may alter the immunogenicity of antigens if the peptide secondary structure is disturbed [55]. Chitosan and carboxymethyl chitosan NP were found to be excellent carriers for oral vaccine delivery of extracellular products of *V. anguillarum* (pathogenic bacteria). The NP prepared were stable in the gastric pH, had sustained release and protected the antigenic protein from entering spleen and kidney which is critical for immune response [46].

## 5. Chitosan in Nasal Drug Delivery

Nasal delivery is a non-invasive technique of delivering drugs to reach the respiratory system, the brain and/or systemic circulation. A significant challenge with the delivery of drugs through the nasal route is the mucociliary clearance of drugs. Moreover, hydrophilic drugs, proteins and peptides, nucleic acids and polysaccharides present difficulties because of their low permeability across the nasal epithelium. Nasal absorption is critical for the drugs to exhibit their action. The physical characteristics of drugs that govern nasal absorption include molecular weight, lipophilicity and charge. The drugs that do not cross the nasal membrane undergo mucociliary clearance. This limitation can be overcome by developing a mucoadhesive system. Chitosan is biodegradable, biocompatible, exhibits low toxicity, adheres to mucus and opens the tight junctions of nasal membrane. Owing to these properties, chitosan has applications in nasal delivery [56]. The three ways of nasal absorption is by transcellular pathway, paracellular pathway and via trigeminal nerves [57]. Several specific examples are given below.

Carbamazepine is used in the treatment of epilepsy and it is very important for the drug to cross the blood brain barrier (BBB). Carboxymethyl chitosan NPs of carbamazepine have found to enhance the bioavailability and brain targeting via the nasal route. The brain-to-plasma exposure ratio was 150% when carbamazepine was administered as chitosan NPs intranasally [25]. In Alzheimer’s disease (AD), a person’s sex is a risk factor and in women with AD, the levels of 17β-estradiol are found to be relatively low. Estradiol, a potent sex hormone, has been used in the prevention and treatment of AD. It is important for estradiol to achieve a sufficient tissue concentration in the brain to exhibit it effect. When estradiol is given orally, the cerebrospinal levels are very low. The cerebrospinal fluid levels of estradiol were found to be high compared to plasma levels when estradiol was administered intranasally as chitosan NPs. These results suggest that estradiol is transported to the brain directly when given through the nasal route as chitosan NPs. As another example, the bioavailability of leuprolide, used in the treatment of prostate cancer and hormone-dependent diseases, was found to be increased when formulated as thiolated-chitosan NPs [57]. Chitosan NPs and thiolated-chitosan NPs of leuprolide were prepared. There was a 2–5-fold increase in drug transport across porcine nasal mucosa when leuprolide was formulated as chitosan NPs or thiolated-chitosan NP, respectively, compared to leuprolide solution. The drug exposure, as measured by area under the plasma concentration vs. time curve AUC, increased by 6.9-fold with thiolated-chitosan NP [58].

## 6. Chitosan in Pulmonary Drug Delivery

Both local and systemic effects can be achieved by drug delivery to the lungs. There are several advantages to delivering drug to the lungs compared to other routes such as rapid and sustained drug delivery, high efficacy and no hepatic first-pass effect. The factors that enhance drug delivery via the lungs are the large surface area of the lungs, high tissue vascularity and the thin absorption barrier [59]. The barriers for drug delivery via the lungs include the bronchial mucus layer, the alveolar lining fluid, epithelial cells, macrophage clearance and proteolytic degradation [60]. In the recent review published by Islam and Ferro [61], drug delivery to the lungs with the help of chitosan based nanoparticles can be achieved. The authors claimed it to be beneficial that for pulmonary drug delivery, the positive charge on the surface of chitosan provides mucoadhesive properties. This adherence to the lung mucosa increases the potential for drug absorption; the positive charge on chitosan has been previously shown to open the intercellular tight junctions of the lung epithelium thereby increasing uptake. Interestingly, chitosan possesses antibacterial activity by binding to phosphoryl groups and lipopolysaccharides on bacterial cell membranes, which is an additional benefit in fighting pulmonary bacterial infections.

A NP dry powder inhalation (DPI) of rifampicin, an antitubercular drug, was formulated with chitosan as the polymer. This NP formulation has shown sustained drug release until 24 h and no toxicity at both cell and organ. An in vivo study of this formulation showed that rifampicin exhibited increased maximal plasma concentration (C_max_), AUC and extended mean residence time (MRT) with the NP formulation [62]. Itraconazole is an anti-fungal drug which, when administered orally, suffers from low solubility. To treat pulmonary infections effectively, itraconazole has to be administered via the pulmonary route. Aerosolization would be an advantage for antifungal agents as it can provide a high drug concentration at the site of action, passive targeting and reduced systemic toxicity. The aerosolization properties of itraconazole can be significantly improved by formulating the drug in spray-dried chitosan NP with lactose, mannitol and leucine. The pulmonary deposition of itraconazole was shown to be increased when formulated as spray-dried microparticles containing itraconazole loaded chitosan NP [63]. Some of the other applications of chitosan in pulmonary drug delivery as depicted in Table 2.

## 7. Mucoadhesion

**Figure 4:**
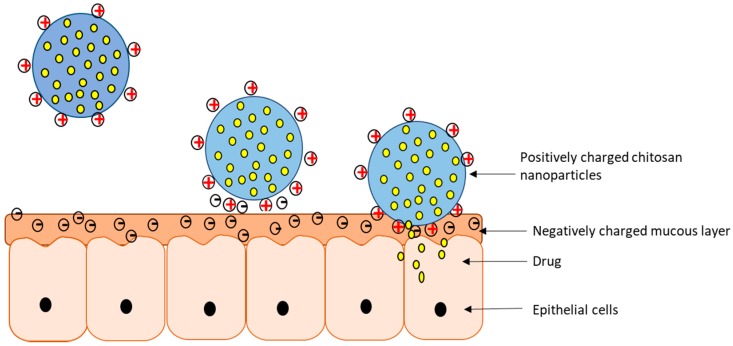
Schematic representation of chitosan loaded nanoparticles (CS-NP) structure and interaction with the mucus layer. From left to right: CS-NP upon reaching the mucosal layer bind to the negatively charged mucus by virtue of electrostatic attraction and release the drug over time.

One of the major drawbacks of delivering proteins/peptides or macromolecules through a non-injection route is the limited absorption at mucosal sites. For local delivery in the GI tract or nasal/buccal cavity or to the vaginal, urethral or pulmonary sites, the drug delivery system should be mucoadhesive and release the drug. Mucoadhesive nanoparticles/microparticles can adhere to the mucus membrane and release the drug over time (Figure 4), with the potential to reduce dosing frequency. Uptake of drugs into the systemic circulation can be achieved by transient and reversible opening of tight junctions in between epithelial cells by certain polymers and chitosan is one such polymer [69]. Some of the other mucoadhesive polymers apart from chitosan are alginate, guar gum, pectin, carrageenan K type II, gelatin, poly (vinyl pyrrolidone), poly (vinyl amine), poly (ethylene glycol) and poly (ethylene oxide) and its copolymers and poly (acrylic acid) and poly (methacrylic acid) derivatives [70].

As discussed earlier, the mucoadhesive property of the chitosan is attributed to its strong positive charge which helps in forming a bond with negatively charged mucus. Mucus is present in the organs of the GIT and the respiratory tract. The GIT is characterized by varying pH and an enzyme environment which makes it difficult for oral delivery of protein/peptide drugs and DNA. Chitosan is an excellent carrier for such drugs as it is mucoadhesive, permeation enhancer and forms a protective barrier for the drug [71]. Particle size also plays a role in the case of chitosan nanoparticles, as smaller particles may be more able to penetrate the mucous layer. Table 3 provides examples where the mucoadhesive property of chitosan is utilized.

### 7.1. Buccal Drug Delivery

Buccal drug delivery involves drug delivery to the buccal cavity and to the systemic circulation. The advantages by delivering drug through buccal route include; increased bioavailability, less amount of drug required and bypassing the hepatic first pass effect. Moreover, the drug need not be exposed to the harsh GI environment [81]. The high permeability of buccal mucosa is a suitable target for novel formulations [82].

Giovino and co-workers have investigated chitosan buccal films of insulin loaded poly (ethylene glycol) methyl ether-block-polylactide (PEG-b-PLA) NP [83]. The NP showed excellent mucoadhesive properties and insulin release from the NP was slow and sustained (70%). Ex vivo studies reveal 1.8-fold enhancement in insulin permeation from NP compared to pure drug. Polysaccharide-based NP of alginate, chitosan and pectin for buccal delivery were prepared and compared. Chitosan NP were not stable in the saliva compared to alginate and pectin NP. Contrary to this, chitosan NP were cytocompatible while alginate and pectin NP have shown cytotoxicity [80]. As another example of buccal delivery, curcumin prepared as polycaprolactone nanoparticles coated with chitosan. As a third example, we can consider the NP encapsulation ofthe enriched flavonoid fraction (EFF-Cg) obtained from *Cecropia glaziovii.* EFF-Cg is used for several purposes such as controlling blood pressure and as a diuretic, antiasthmatic and hypoglycemic agent but it suffers from low bioavailability. EFF-Cg loaded PLGA nanoparticles were prepared for buccal delivery in the form of chitosan films. The bioavailability of EFF-Cg was improved and no signs of cytotoxicity were seen [84]. Thus, addition of chitosan in this hybrid approach has enabled both a high NP encapsulation efficiency and a stronger interaction with mucus [84]. Although the above-mentioned applications of chitosan are promising, chitosan NP suffers from stability issues in oral environment. 

### 7.2. Site Specific Delivery in the GIT

Due to its properties of mucoadhesivity, permeation enhancement, biocompatibility, biodegradability and efflux pump inhibition, chitosan is one of the most suitable polymers for oral drug delivery [81]. In the above section on oral drug delivery with chitosan formulations, there are a few examples of mucoadhesive drug delivery systems there were formulated to release drug in the small intestine [40,41]. Alternatively, Suvannasara et al. attempted to enhance the mucoadhesive property of chitosan in the acidic environment (stomach) by conjugating C2-N position of chitosan with aromatic sulfonamide, 4-carboxybenzenesulfonamide-chitosan (4-CBS-chitosan) [85]. The 4-CBS-chitosan has shown higher mucoadhesion compared to pure chitosan. Moreover, the swelling ratio was higher than the pure chitosan suggesting that the polymer can tolerate acidic stomach conditions. The authors here recommend using 4-CBS-chitosan for delivering drugs specifically to the stomach (drugs with absorption window in stomach).

For the treatment of colonic diseases such as ulcerative colitis, Chron’s disease, irritable bowel disease and colon infection, colon specific drug delivery is more appropriate. Several polymers have been investigated for colon specific drug delivery [86]. Of the several natural polymers available, chitosan is best suited for colon specific delivery owing to the properties of biodegradability by enzymes in colon, chelating ability and biocompatibility [87]. Chitosan-vancomycin NPs for colon delivery were prepared by two different methods; ion gelation and spray drying. The NPs prepared by spray drying have shown high encapsulation efficiency and better drug release compared to the NPs made by ion gelation [83]. Coco et al. have compared the ability of NPs made with chitosan to other polymers for inflamed colon drug delivery [88]. Several batches of NPs were prepared by entrapping ovalbumin (OVA) into Eudragit S 100, trimethylchitosan, PLGA, PEG-PLGA and PEG-PCL, separately. Of all the NPs made, NPs with trimethyl chitosan have shown the highest permeability of OVA. However, a high permeability was also seen with PEG-PLGA NPs as they were coated with mannose for active targeting of the area of inflammation. As another example, chitosan-carboxymethyl starch nanoparticles of 5-aminosalicylic acid, another drug for inflammatory bowel disease, have been prepared which achieved high entrapment efficiency as well as controlled drug release [89].

Although chitosan has shown its ability to deliver drug to the colon, there are a few issues to be addressed such as toxicity studies in humans, the impact of the GI tract inflammation characteristic of these disorders on mucoadhesion and drug absorption, the stability of these compounds in that biological environment and achieving a manufacturing scale of fabrication [90]. Demonstration of superiority to the Eudragit polymers currently in use for colon specific drug delivery of specific drugs will also be required [91].

Some of the other drug delivery routes have been explored for mucosal delivery with chitosan are vaginal, nasal, pulmonary and ocular. Systemic absorption of insulin has been demonstrated by formulationin chitosan NPs and administration by the nasal route. Insulin loading up to 55% was achieved and nasal absorption of insulin was greater from chitosan NP [92]. Rosmarinic acid loaded chitosan NP have been prepared by an ion gelation method for ocular delivery. The NPs showed no cytotoxicity against the retinal pigment epithelium (ARPE-19) nor the human cornea cell line (HCE-T). The permeability of rosmarinic acid facilitated by the NP formulation was found to be significantly higher compared to free solution. Mucoadhesion studies reveal that the NPs interact with the ocular mucosa [93]. Imiquimod was formulated as both chitosan coated PCL nanocapsules embedded in hydroethylcellulose gel and PCL nanocapsules embedded in chitosan hydrogel for vaginal delivery to treat human papillomavirus infection [94]. The former was found to show higher mucoadhesion while the latter has shown high drug permeation. Balancing the considerations of mucoadhesion, permeation and drug retention, the latter was selected as the best delivery system [94].

## 8. Pharmacokinetics (PK) of Chitosan Based Formulations

The pharmacokinetics of chitosan-based NPs is similar to those of other polymeric NPs because the same principles of drug release apply as discussed above. The most important property of chitosan to be exploited is its mucoadhesion. In the following section, we explore the pharmacokinetic (PK) features of chitosan-based NP. A PK study was done in beagle dogs to assess the bioavailability of cyclosporin-A (Cy-A) encapsulated into NPs comprised of chitosan, gelatin-A or sodium glycocholate (SGC). A control group received the standard oral micro-emulsion formulation (Neoral^®^). The C_max_ was markedly increased in the case of both the chitosan and gelatin NP formulations while the C_max_ decreased with SGC NPs as compared to Neoral. There was a 2.6-fold increase in the AUC of Cy-A from chitosan NPs compared to SGC NPs and 1.8-fold increase in AUC from gelatin NPs compared to SGC NPs. The relative bioavailability of Cy-A from SGC NPs was decreased by 36% when compared to marketed formulation. This could be due to the negative charge of the SGC NPs which could have hindered the NPs from adhering to the intestinal mucus and thus may have reduced the drug absorption across the intestinal epithelium. This supports the idea that a positive charge on chitosan NPs can help in mucoadhesion and increase in relative bioavailability, improved by 73% in this case [45].

**Figure 5:**
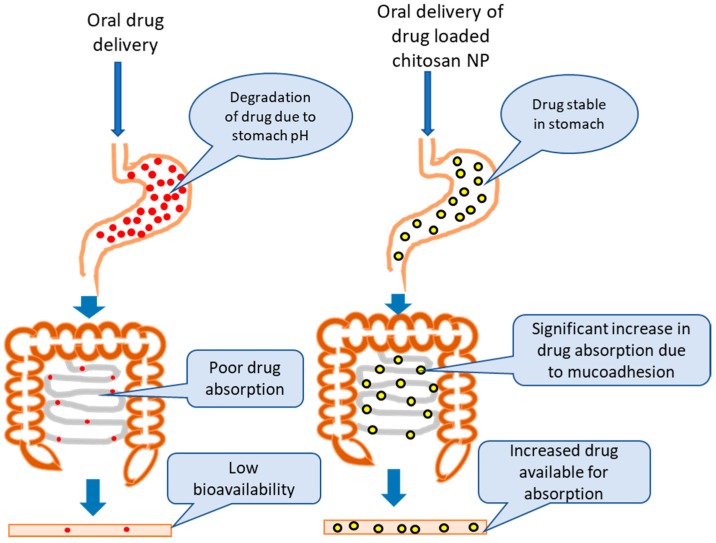
In vivo efficiency of drug loaded chitosan NPs in enhancing the drug absorption via the intestinal epithelium thereby increasing the drug available for absorption.

Chitosan based NPs of Bay 41-4109 were developed with the primary goal of prolonging circulation time of the drug in blood. A PK study in rats demonstrated a 3.3-fold increase in C_max_, increased AUC and 4-fold increase in absolute bioavailability of chitosan NPs compared to the Bay 41-4109 suspension. The enhanced intestinal absorption of Bay 41-4109 can be attributed to either increased interaction between the positive charge of chitosan with the negative charge of cell membranes or the mucoadhesive character of chitosan NPs, enabling them to release drug over time in the small intestine [44]. Enoxaparin has little to no oral bioavailability. In order to facilitate oral bioavailability, enoxaparin-loaded alginate-coated chitosan NPs (Enx-Alg-CS-NPs) were formulated, resulting in a 3-fold increase in AUC for oral enoxaparin (50 mg/kg in rats) and representing 20% of the AUC achieved with intravenous dosing (1 mg/kg). The increased intestinal permeation of enoxaparin in rats could be due to enhanced paracellular transport of the drug across intestinal epithelium owing to the mucoadhesive property of chitosan [47]. Chitosan NPs have many applications in increasing the oral bioavailability and the in vivo efficiency, illustrated in the following schematic representation (Figure 5).

## 9. Toxicity and Safety of Chitosan

Chitosan is biodegradable and the process occurs either by chemical or enzyme catalysis. Degradation of chitosan is dependent on the degree of deacetylation and the availability of amino groups. Additionally, chitosan is approved as safe by US-FDA and EU for dietary use and wound dressing applications. However, the toxicity of chitosan increases by increasing charge density and degree of deacetylation [37]. As of this writing, we have not found any published data showing human toxicity of chitosan based formulations or questioning the safety of chitosan for human use. However, there are several animal toxicity studies reporting good safety in vivo and in vitro.

Aluani et al. have reported an in vivo toxicity study of two types of quercetin-loaded chitosan NPs (QR-NP1, QR-NP2) in male Wistar albino rats [56]. Briefly, the rats were divided into six groups and treated with saline, quercetin, empty NP or quercetin NPs in two related formulations. The rats were sacrificed, livers were collected, antioxidant defense marker (malondialdehyde (MDA) and glutathione (GSH)) levels were assessed. Oral administration of empty and quercetin loaded chitosan NPs indicated no change in body weight, relative rat liver weights, liver histology and hematology and biochemical parameters. There was no increase in MDA levels with both empty and quercetin loaded chitosan NP. GSH levels in animals with one of the NP formulations were slightly decreased. This data was also supported by in vitro cytotoxicity study which concludes chitosan NPs as safe carrier for quercetin in oxidative stress associated injuries.

Several airway-based cell culture models such as bronchial Calu-3 and alveolar 549 cells are in use to demonstrate the safety and toxicity of chitosan-based formulations for pulmonary drug delivery [61]. Lung toxicity of these biodegradable NPs was evaluated in mice in vivo [95]. Three NP formulations, PLGA NPs coated with chitosan, poloxamer 188 NPs and PVA NPs, were analyzed for biodistribution and inflammatory potential after a single dose administration in mice. Analysis of brancheoalveolar lavage cell population, protein secretion, cytokine release and histopathology were carried out. Chitosan-coated PLGA NPs showed better biodistribution and lower toxicity compared to non-coated NPs. Low levels of cytokine release indicate that chitosan coated PLGA NPs did not induce an inflammatory response. Chitosan-coated PLGA NPs showed a favorable biodistrbution as well [91]. Grenha et al. 2007 reported absence of toxicity in vitro with Calu-3 cells and A549 epithelial cells with NP concentrations up to 1.3 mg/mL [96].

In vitro cytotoxicity of chitosan based NP against buccal cells (TR146) was evaluated by Pistone et al. [79]. Three types of NP were prepared; alginate, Zn^+2^—crosslinked pectin NP and chitosan NP crosslinked with TPP. An in vitro cytotoxicity test showed favorably that chitosan NPs were less cytotoxic compared to the alginate and pectin NPs. Moreover, chitosan alone was found to be more cytotoxic than the chitosan NP which could be due to the linker attached to chitosan NP or the intracellular processing response differential to free material vs NPs. The cytotoxicity of chitosan NP was shown to be further reduced either by increasing the concentration of the linker (TPP) or using chitosan with lesser degree of deacetylation.

## 10. Clinical Vaccine Trials of Chitosan Based Formulations

While clinical use of oral or mucoadhesive drug formulations containing chitosan remain on the horizon, there are already human vaccines in development which use chitosan as an adjuvant. CRM_197_, a diphtheria toxoid antigen formulated as a chitosan-glutamate intranasal system, was evaluated in healthy human volunteers for the safety and immunostimulatory effects. Three groups of volunteers received intranasal diphtheria vaccine (500 µg of CRM_197_ with 9.5 mg of chitosan glutamate 213 and 2.5 mg mannitol), antigen alone (Day 0 and 28) and alum-adsorbed diphtheria toxoid vaccine via IM injection (Day 0). Serum IgG and IgA were collected on Days 27 and 42. Results suggest that chitosan was well tolerated with only minor adverse effects such as nasal discharge, blockage and discomfort. Antibody levels of the group receiving CRM_197_ and chitosan intranasally increased after the second (booster) vaccination [97].

An intranasal vaccine (NV-VLP) was formulated as spray dried powder composed of chitosan and norovirus VLP antigen with monophosphoryl lipid (MPL) as immune enhancer. This was tested in Phase 1 clinical studies, two randomized, double blinded, controlled studies with healthy volunteers. In one study, 5, 15 and 50 µg of antigen and chitosan alone were evaluated. In another trial, four groups of healthy volunteers were given MPL/chitosan (500 or 100 µg VLP per dose), chitosan only and placebo. Symptoms were recorded for a week after vaccination and safety evaluation up to 180 days. No vaccine related adverse effects were seen and significant immune response was seen with 100 µg dose. These results reveal that intranasal administration of vaccine may induce IgA secretion from intestinal mucosal tissues [98]. Similar such work has been done in healthy volunteers which provide further evidence of the efficacy of this vaccine approach [99].

## 11. Preparation of Chitosan Nanoparticles

Ionotropic gelation, microemulsion, emulsification solvent diffusion and emulsion based solvent evaporation are the most common methods to prepare chitosan-based nanoparticles. Usage of less organic solvent and lesser force are some of the main advantages most of these methods offer. The key characteristics that are found to affect the particle size and surface charge of chitosan NPs prepared by these methods are molecular weight and the degree of acetylation of the chitosan. Some of the mechanisms by which drugs are entrapped within the polymeric matrix are electrostatic interaction, hydrogen bonding and hydrophobic interactions. Drug loading and release are not the only key features, however. The intended use of the nanoparticles and the physiological environment at the site of administration must be taken into account, e.g. not only ionic strength, or the presence of salts, enzymes and proteins but also pH stability (consider the milieu of the eye vs. the GI tract). Fortunately, we now have multiple formulation methods to choose from.

### 11.1. Ionotropic Gelation

This is a simple technique where the chitosan solution (positively charged) is dissolved in acetic acid or any polyanionic solution (negatively charged) with or without a stabilizing agent such as poloxamer. Nanoparticles are readily formed due to complexation between positive and negative charged species during mechanical stirring at room temperature, resulting in separation of chitosan in spherical particles of different sizes and surface charges. Generally, the reported particle size ranges from 20 to 200 and 550 to 900 nm. Chitosan-TPP/vitamin C nanoparticles were prepared via ionotropic gelation between the positively charged amino groups of chitosan-TPP and the vitamin C, with constant stirring at room temperature for just 1 h [100,101]. A few advantages of ionotropic gelation include: the processing conditions are mild and it uses an aqueous environment, low toxicity and little chance of altering the chemistry of the drug to be encapsulated. The main disadvantages of this method are its poor stability in acidic conditions and difficulty in entrapping high molecular weight drugs [2,102].

### 11.2. Complex Coacervation Method

Coacervation is a technique of separating spherical particles by mixing electrostatically driven liquids. DNA-chitosan nanoparticles are formed by coacervation of the positively charged amine groups of chitosan and the negatively charged DNA phosphate groups [103,104]. Entrapment efficiency and drug release are governed by the molecular weight of the two polymers [105,106]. An advantage of complex coacervation is that the process can be entirely performed in an aqueous solution at low temperature. This provides a better chance of preserving the activity of the encapsulated substances. The main disadvantages of this method are the poor stability of the NPs, low drug loading and crosslinking of the complex by a chemical reagent such as toxic glutaraldehyde is necessary [101,107,108]. In the polyelectrolyte complex (PEC) method, an anionic solution (for example, dextran sulfate DNA solution) is added to the cationic polymer (e.g., chitosan solution dissolved in acetic acid, gelatin and polyethylenimine), under mechanical stirring at room temperature followed by charge neutralization. Advantages include: the method is simple, there is an absence of harsh conditions and the nanoparticles form spontaneously [2,101]. Low molecular weight water-soluble chitosan (LMWSC) nanocarriers were developed by the PEC method for insulin, resulting in insulin-loaded chitosan NPs with a reported mean diameter of ~200 nm and sustained release profile in vitro [101].

### 11.3. Coprecipitation Method

The addition of a chitosan solution, prepared in low pH acetic acid solution, to a high pH 8.5–9.0 solution, such as ammonium hydroxide, results in coprecipitation and the formation of a highly monodisperse nanoparticle population. Nanoparticles of diameters as low as 10 nm can be prepared with high encapsulation efficiency [101,107]. The wide range of particle size is seen with this method which could be a disadvantage. A coprecipitation method was used to prepare lactic acid-grafted chitosan (LA-g-chitosan) nanoparticles where ammonium hydroxide was used to form coacervate drops. This method yielded spherical and uniformly distributed nanoparticles [109].

### 11.4. Microemulsion Method

In this method, chitosan in acetic acid solution and glutaraldehyde are added to a surfactant in an organic solvent such as hexane. This mixture is kept on continuous stirring at room temperature, allowing the nanoparticles to form overnight as the cross-linking process is completed. Organic solvent is then removed by evaporating under low pressure. The product at this point has excess surfactant which can be removed by precipitating with calcium chloride followed by centrifugation. The final nanoparticle suspension is then dialyzed and then lyophilized [110]. A very narrow size distribution is seen with this method and the size can be controlled by the concentration of glutaraldehyde in the preparation of the NPs. This method results in formation of small sized nanoparticles [111]. Some disadvantages with this method include usage of organic solvent, a lengthy process and a complex washing step [2].

### 11.5. Emulsification Solvent Diffusion Method

An o/w emulsion is prepared by mixing organic solvent into a solution of chitosan with stabilizer under mechanical stirring followed by high pressure homogenization [45,112]. Size range of 300–500 nm could be achieved with this method. Polymer precipitation occurs when a large amount of water is added to the emulsion, forming nanoparticles. This method is best suited for entrapment of hydrophobic drugs, for which the entrapment efficiency is found to be high. The major disadvantage of the method includes usage of high shear forces.

### 11.6. Emulsion Based Solvent Evaporation Method

This method is a slight modification of the above method but avoids high shear forces. Particle size of below 300 nm can be achieved with this method. An emulsion is prepared by adding organic solvent to a solution of chitosan with surfactant followed by ultrasonication. The emulsion formed is then added to a surfactant solution and allowed to stir until the organic solvent is evaporated, forming nanoparticles. The NP are then washed and centrifuged multiple times to remove excess surfactant followed, by lyophilization to achieve freeze-dried nanoparticles [113,114].

### 11.7. Reverse Micellar Method

The surfactant is dissolved in an organic solvent followed by the addition of chitosan, drug and crosslinking agent, under constant overnight vortex mixing. The organic solvent is evaporated results in formation of transparent dry mass, then the latter is dispersed in water and then a suitable salt is added for precipitating the surfactant [115,116]. A very narrow size range nanoparticle is seen and organic solvents are used [117]. Doxorubicin-dextran conjugate loaded chitosan nanoparticles were prepared by reverse micellar method. The surfactant used in this method was sodium bis (ethyl hexyl) sulfosuccinate (AOT) was dissolved in n-hexane. The NP are formed by adding liquid ammonia and 0.01% glutaraldehyde to AOT solution, 0.1% chitosan in acetic acid, doxorubicin–dextran conjugate upon continuous stirring at room temperature [118,119].

## 12. Limitations

Chitosan has low solubility in neutral and alkaline pH. For GI applications, its mucoadhesion and permeation enhancer properties are strongest in the duodenal area, which can be modulated with chitosan derivatives. The toxicological profile of chitosan derivatives is still under investigation. There are multiple preparation methods now available for chitosan nanoparticles but formulators will have to adapt methods to suit the physicochemical properties of the specific drug to be encapsulated, with a careful choice of a specific chitosan in terms of its molecular weight and degree of acetylation and consideration of chemical modification. To date, chitosan has shown little or no toxicity in animal models and there have been no reports of major adverse effects in healthy human volunteers but clinical data are lacking. Even though chitosan is approved in dietary use, wound dressing applications and cartilage formulations, as of this writing there is not yet a chitosan-based drug formulation approved for mass marketing [120]. Issues regarding scale up of fabrication methods will likely be informed by that of other polymeric formulations.

## 13. Conclusions and Future Work

Based on the versatility of chitosan, it has many potential applications in drug delivery via the GIT, nasal, pulmonary routes as explained in this review. Chitosan NP can effectively deliver drug at specific sites by retaining the drug locally to permit an extended time for drug absorption. Mucoadhesion and absorption enhancement of chitosan makes it possible to deliver drugs directly from the nose to the brain. Similarly, lung infections and colon diseases can be effectively targeted locally with chitosan NP. A chitosan-based nasal formulation of morphine (Rylomine^TM^) is currently in Phase 2 clinical trials (UK and EU) and Phase 3 clinical trials in the U.S. We anticipate that when it reaches the market it will pave way for similar products in the near future as well as assist in discerning any unanticipated effects in humans [120]. And, while not specifically addressed herein, we look forward to additional advances in the use of chitosan nanoparticles in targeted cancer theranostics, dermatologic applications and targeted parenteral drug delivery systems [121,122,123,124]. With the advent of new strategies in overcoming the limitations of chitosan by improved formulation methods for a wider variety of drugs and even macromolecules, we expect to see more chitosan research work in near future, especially in nasal and pulmonary drug delivery. We hope that future work on chitosan nanoparticles prepared by chitosan or chitosan derivatives will also focus on toxicity studies in humans.

## Figures and Tables

**Table 1:** Applications of chitosan nanoparticles in oral drug delivery.

Drug	Composition	Purpose	Research Findings	In Vivo	Reference
Tamoxifen citrate	Chitosan, soybean lecithin NP	Intestinal permeation of tamoxifen in lecithin/chitosan NPs through the rat intestinal wall.	Ex vivo experiment using a jejunum chamber from the small intestine of male Wistar rats. Lecithin/chitosan nanoparticles improved the non-metabolized drug passage across the rat intestinal tissue.		[40]
Sunitinib	Chitosan	Biophysical characterization	1. In vitro biodegradation was improved in the presence of lysozyme. 2. In vitro controlled release was demonstrated with 69% released within 72 h.		[42]
Hydro phobic Bay41-4109	LMW chitosan	Solubility and bioavailability enhancement.	1. In vitro drug release studies showed a sustained release profile. 2. Low cytotoxicity was demonstrated in a liver cell line.	In vivo studies were performed in rats. The absolute bioavailability of Bay41-4109 NPs was >70%, nearly 4-fold higher than other formulations	[44]
Insulin	Chitosan, TPP	Preparation, characterization and stabilization of insulin—chitosan NPs.	1. In vitro validation-Caco2 and co-culture: Caco-2 cells internalized the NPs effectively.	In vivo studies where decreased glycaemia was observed in diabetic rats after insulin NP administration.	[43]
Cyclosporin-A	Chitosan HCl, Poloxamer 188, sodium glycolate, gelatin, soya lecithin	To develop and characterize Cy-A as positive charge NPs to improve GI uptake, bioavailability	Cy-A encapsulation efficiency was very high at 88–94%.	In vivo studies in beagle dogs showed relative bioavailability of Cy-A was significantly increased by NPs.	[45]
Extra cellular products (ECPs) of *Vibrio anguillarum* (Protein carrier)	Chitosan carboxymethyl chitosan	To study the efficacy of chitosan NPs as a vehicle for oral antigen delivery in fish vaccination.	In vitro release study was performed in Tris-buffer (pH 2.0, 4.5) & PBS (pH 7.4); the highest cumulative release was 58% at pH 7.4 followed by 37% at pH 2.0.	Biodistribution showed NP uptake in spleen and kidney. Lysozyme and complement evaluation. It showed elevated specific antibody and higher concentrations of lysozyme activity and complement	[46]
Alendro-nate sodium	Chitosan LMW, sodium tripolyphosphate (TPP), fluorenyl-methyloxycarbonyl chloride (FMOC)	To study the influence of physical parameters on drug encapsulation efficiency	In vitro drug release was performed in 0.1N HCl and PBS (pH 6.8). The NPs released alendronate faster in 0.1N HCl compared to PBS. Encapsulation efficiency was ~80%.		[28]
Catechins (+) catechin (C) and (−) epigallocatechingallate (EGCg)	Chitosan LMW, sodium tripolyphos-phate, tris[2-carboxyethyl] phosphine hydrochloride (TCEP)	To enhance the intestinal absorption by encapsulation of C, EGCg in chitosan NPs	Ex vivo study using a jejunum chamber from mice. The cumulative amounts transported after encapsulation were significantly higher for C and EGCg, respectively.		[39]
Enoxaparin	Chitosan, STPP, sodium alginate	Alginate coated chitosan-NPs containing enoxaparin for oral controlled release	In vitro drug release showed only 2% drug release in SGF (pH 1.2) but 60% in SIF (pH 6.5).	In vivo studies were performed in albino rats: the oral bioavailability of enoxaparin in Alg-CS-NPs bioavailability was significantly higher compared to enoxaparin solution. A 60% reduction was seen in thrombus formation in rat venous thrombosis model.	[47]
Scutellarin	Chitosan, deoxycholic acid, vitamin B12	Enhancement of scutellarin oral delivery efficacy by Vit B12 modified amphiphilic chitosan derivatives to treat type II diabetes-induced retinopathy	Cytotoxicity study of Chit-DC and Chit-DC-VB12 displayed low cytotoxicity in Caco-2 cells.	In vivo studies: 1. Zebra fish embryo: development of the embryo was unaffected 2. Sprague-Dawleys rats: the AUC of scutellarin NP was 2–3 fold higher than free scutellarin and efficacy was achieved	[48]
Fucoidan	Chitosan, Tc-methylene di-phosphonate	To prepare and evaluate pH sensitivity of CS/F NPs	In vitro drug release of Tc-MDP from CS/F NPs rose as the pH levels changed from 2.5 to 7.4. CS/F NPs was stable into the stomach and decompose in the intestine.		[49]
Tolbut-amide	Chitosan, PLGA, streptozotocin	To prepare PLGA NPs modified with chitosan to form TOL-CS-PLGA NPs to improve bioavailability and reduce dose frequency.	In vitro drug release of TOL-CS-PLGA NPs showed sustained release in PBS, pH 7.4. Cytotoxicity study of TOL-CS-PLGA NPs were non-toxic in HePG2 cells.	In vivo study was performed in adult Sprague-Dawley rats: the TOL-CS-PLGA NPS showed a long-acting hypoglycemic effect over 8 h, significantly longer than metformin tablets.	[50]
Gemcita-bine	Chitosan LMW, penta sodium tripolyphos-phate	To prepare gemcitabine-loaded chitosan NPs (Gem-CS-NP) for oral bio-availability enhancement	1. In vitro drug release of Gem-CS-NPs showed controlled release by a two-phase process. 2. Absorption studies in an intestinal sac model: the absorption of Gem-CS-NPs intestinal transport increased 3–5-fold compared to free drug.		[51]
Naringenin	Sodium alginate, chitosan, streptozotocin	To prepare alginate coated chitosan core shell NPs for effective oral delivery	In vitro drug release from NPs was 15% in SGF (pH 1.2) and 90% in pH 7.4 in slow, sustained fashion.	In vivo study in rats showed lack of toxicity; and an anti-diabetic effect: naringenin NPs have better efficacy in lowering blood glucose levels compare to free drug.	[52]
Epigallocatechin gallate (EGCG)	*N*-carboxymethyl chitosan (MW = 61 kDa), chitosan hydrochloride	To develop EGCG-chitosan/β-Lg NPs to achieve a prolong release for oral administration in the GI tract	In vitro drug release and degradation of EGCG-chitosan/β-Lg NPs was slower in simulated stomach condition compare to control particles		[53]
Quercetin	Chitosan, sodium alginate, sodium pyruvate, l-glutamine	To develop and evaluate quercetin-chitosan/alginate NPs to preserve its antioxidant property without causing systemic toxicity.	In vitro cytotoxicity of both empty NPs and quercetin NPs exhibited nontoxic behavior in HepG2 liver cells when exposed for a period up to 72 h.	In vivo toxicity study in Wistar rats displayed no change in body weight, rat liver weight, histology, hematology and biochemical parameters after oral administration of empty NPs and quercetin loaded NPs.	[54]

**Table 2:** Applications of chitosan nanoparticles in pulmonary drug delivery.

Drug	Composition	Purpose	Research Findings	In Vivo	Reference
Rifampicin	TPP, lactose, Tween 80	Preparing CS-NPs dry powder to achieve local and sustained targeting of anti-tubercular drugs in order to reduce dosage and frequency	1. In vitro release showed 90% release of RFM from CS-NPs within 24 h. 2. Cell viability of J774 macrophage cells showed negligible toxicity of RFM-NPs up to 12 h exposure at concentrations up to 0.5 mg/mL	In vivo study—male Wistar rats. A marked increase in Cmax, t1/2 and AUC was seen in RFM-NPs compared to other formulations.	[62]
Itraconazole	Hydroxypropyl-beta-cyclodextrin (HPβCD), mannitol, lactose, TPP, l-leucine	To develop chitosan NPs for pulmonary delivery of itraconazole as a dry powder formulation	1. Encapsulation efficiency of 55% was obtained in 1:3 ratio of chitosan:TPP. 2. In vitro drug release was ~80% during the first 4 h and the remaining encapsulated drug was released over 48 h.		[63]
Baclofen and siRNA	*N*,*N*,*N*-tri-methyl chitosan, TPP	To prepare and evaluate baclofen-trimethyl chitosan/TPP NPs (Bac-TMC/TPP NPs) in a dry powder formulation	1. Low in vitro cytotoxicity of TMC and Bac-TMC in A549 cells when treated with four different polymers for 48 h. 2. Cellular uptake of Bac-TMC3/TPP/siRNA NPs was greatly enhanced by clathrin -mediated cellular uptake pathway.		[64]
Heparin (LMWH)	Chitosan, lipoid S100, glycol chitosan	To prepare and evaluate LMWH chitosan and glycol chitosan NPs for enhancing the pulmonary absorption of LMW heparin.	In vitro drug release of lipoid S100-LMWH GCS NPs in SLF showed progressive LMWH release up to 6 h, followed by a plateau for 24 h.	In vivo studies were performed in mice: the aerosol-type administration of free LMWH and Lipoid S100-LMWH GCS NPs led to a significant elongation of the coagulation time	[65]
Theo-phylline	Chitosan thioglycolic acid, TPP	To develop and evaluate whether theophylline-thiolated chitosan NPs can enhance theophylline’s capacity to alleviate allergic asthma	In vitro mucoadhesive study of TCNs exhibited a gradual increase in mucin binding and adsorption for 12 h compared to unmodified chitosan	In vivo study was performed in mice and the anti-inflammatory effects of theophylline were markedly enhanced when the drug was delivered by TCNs compared to unmodified chitosan or theophylline alone.	[66]
Leuprolide	Thiolated chitosan	To prepare and evaluate leuprolide thiolated-CS-NPs to enhance the half-life and bioavailability of leuprolide via nasal administration	In vitro drug release of leuprolide from thiolated chitosan showed slow and sustained release of drug about 43% in 2 h.	In vivo study was performed in male Sprague-Dawley rats showed improved nasal bioavailability of leuprolide thiolated NPs calculated based on AUC (0–6) was about 19.6% as compared to leuprolide solution alone 2.8%.	[60]
Estradiol (E2)	Chitosan, methylated β-cyclodextrin, TPP	To prepare estradiol-chitosan NPs for improving nasal absorption and brain targeting		In vivo study was performed in male Wister rats: The plasma concentration of E2 from E2-CS-NPs was significantly lower in intranasal administration compare to IV but CSF concentrations of E2 from E2-CS-NPs was significantly higher for intranasal administration compare to IV.	[67]
Tetanus toxoid (TT)	LMW Chitosan, TPP, trehalose	To prepare tetanus toxoid chitosan nanoparticles (TT-CS NPs) as a new long-term nasal vaccine delivery vehicle	In vitro drug release of the TT from CS-NPs showed a rapid release over first 2 h followed by slow release for up to 16 days.	Intranasal immunization with two doses of TT-CS NPs in mice: The results showed the titers were significantly higher for the TT-loaded particles than for the free toxoid and at post-administration of TT-CS NPs IgA levels were significantly higher than the fluid vaccine.	[68]

**Table 3:** Applications of chitosan nanoparticles in mucoadhesive drug delivery.

Drug	Composition	Purpose	Research Findings	In Vivo	Reference
Doxorubicin HCL (DOX)	Chitosan (MW = 400 kDa), 4-CBS, TPP, 1-ethyl-3-(3-dimethylaminopropyl) carbodiimide HCl (EDAC)	Preparation, characterization, in vitro drug release, Topo II inhibitor activity and evaluation of DOX-loaded 4-CBS-chitosan/PLA nanoparticles.	1. In vitro drug release of DOX loaded 4-CBS-chitosan/PLA nanoparticles showed sustained release up to 26 days. 2. Cytotoxic study (MTT) of DOX loaded nanoparticles showed the lowest effect on the cell viability of HepG2 compared to SW620, KAT03 and CHAGO cell lines. 3. Over 72% inhibition of Topo II.		[72]
Alpha-mangostin (AP)	Chitosan (MW = 600 kDa), methane-sulfonic acid, oleoyl chloride, sodium bicarbonate, glycidyl-trimethyl ammonium chloride	To develop a mucoadhesive oleoyl-quaternized chitosan coated nanostructure lipid carrier (NLC) for potential oral administration with enhanced mucoadhesion	In vitro cytotoxicity of AP-NLC and CS-AP-NLC exhibited higher toxicity against Caco-2 than Hela cells.	In vivo toxicology study was performed in zebrafish embryos.	[73]
Cetirizine	Chitosan, 1-ethyl-3-(3-dimethylaminopropyl) carbodiimide hydrochloride (EDC. HCl), *N*-hydroxyl succinimide	Preparation and characterization of mucosal adhesion and drug release of cetirizine-chitosan NP	1. In vitro, the cumulative release of cedH from cedH:CS-NPs and cedH:CTZ-CS-1-NPs were 71% & 76% in absence of lysozyme and increased to 77% & 84% in presence of lysozyme. Burst release and lysozyme induced sustained release was achieved. 2. In vitro cytotoxicity of cedH:CTZ-CS-1 NPs showed non-toxicity in L929 cells and biocompatibility with RBCs.		[74]
5-amino levulinic acid	Chitosan, lactic acid.	To develop and characterize chitosan-based 5-ALA mucoadhesive film to enhance its retention in oral mucosa	In vitro permeation and retention of 5-ALA (1.0% or 10%) were increased. However, 10% 5-ALA exhibited highest values 4 and 17 times, respectively, compared to propylene glycol vehicle.		[75]
Curcumin	Chitosan Low (CLS 50,000–190,000 Da), medium (CSM 190,000–310,00 Da), high (CSH 310,000 to >375,000 Da), polycaprolactone, glycerol	Preparation of mucoadhesive films containing curcumin-loaded NPs to prolong the residence time of the dosage form in the oral cavity and to increase drug absorption through the buccal mucosa	1. Swelling studies for mucoadhesive films containing curcumin loaded NPs showed good hydration in simulated saliva. 2. In vitro release of CSHG5-NP and CSMG5-NP exhibited high release rate approximately 3.4% and 2.8% curcumin respectively, over 24 h.		[76]
Propranolol HCl	Chitosan, TPP, Carbopol 940, poloxamer 407.	To develop a propranolol-chitosan NPs transdermal gels to improve the systemic bioavailability of the drug.	1. In vitro drug release was performed in buffer solution, only 7% and 11% of propranolol was released in 24 h from nanoparticle suspension and gel. 2. Ex vivo drug release was performed in pig ear, skin showed a slow permeation rate from nanoparticles in gel over 24 h.		[77]
C-glycosyl flavonoid enriched fractions of cecropia glaziovii (EFF-Cg)	Resomer PLGA, ploxamer 188, sorbitan monoaleate, chitosan	To develop and characterize EFF-Cg nanocomposites chitosan film containing PLGA NPs.	In vitro cytotoxicity study was performed in Vero cell line: chitosan film and nanocomposite film exhibited low toxicity.		[78]
Alginate and pectin	Chitosan, TPP, Triton X-100	Preparation of alginate and pectin chitosan NPs for oral drug delivery	1. Cytotoxicity was performed in TR146 cells: Chit-NP showed cytocompatibility while Alg-NP, Pec-NP exhibited cytotoxicity at some concentrations. 2. Stability of NP in simulated salivary fluid: The Alg-NP were the most stable (a > 2 h), while Pec-NP and especially Chit-NP were unstable.		[79]
Insulin	Chitosan MMW, PEG, PVP, trehalose	To develop and characterize chitosan films with insulin loaded PEG-b-PLA NPs	In vitro drug release of both insulin NPs showed pH dependent classic biphasic sustained release of protein over 5 weeks and insulin encapsulation efficiency of 30–70%.		[80]
